# Effectiveness of nursing interventions on the sexual quality of life of patients with breast cancer: A systematic review and meta-analysis

**DOI:** 10.1371/journal.pone.0277221

**Published:** 2022-11-03

**Authors:** Jia Lu, Xiao Min Chen, Kai Hong Xie

**Affiliations:** 1 School of Nursing, Zhejiang Chinese Medical University, Hangzhou, China; 2 Nursing Department, Zhejiang Provincial People’s Hospital, Hangzhou, China; Shuguang Hospital, CHINA

## Abstract

**Background:**

Although many studies have reported the effectiveness of nursing interventions on the sexual quality of life of patients with breast cancer, the results have not been synthesized. This study aims to assess the effectiveness of nursing interventions on the sexual quality of life of patients with breast cancer.

**Review methods:**

A comprehensive search was conducted in 11 databases from inception to October 7, 2021. Studies evaluating the effects of nursing interventions on sexual quality of life were included. Study selection, data extraction, and risk of bias assessment were performed by two independent reviewers.

**Results:**

This review pooled 38 studies with 3,664 participants. Meta-analysis results showed that nursing interventions significantly improved sexual quality of life, including sexual function (standardized mean difference [SMD] = 0.98, 95% confidence interval [CI] = [0.60–1.37], P < 0.001) and sexual satisfaction (SMD = 0.99, 95% CI = [0.41–1.57], P < 0.001). In addition, depression (SMD = −1.16, 95% CI = [−2.08–−0.24], P = 0.01) and general quality of life (SMD = 0.20, 95% CI = [0.08–0.33], P = 0.002) were significantly improved, but body image (SMD = 0.17, 95% CI = [−0.08–0.41], P = 0.19) and anxiety (SMD = −0.45, 95% CI = [-0.93–0.02], P = 0.06) did not significantly improve. Subgroup analysis showed that nursing interventions had a stronger long-term effect on sexual function (SMD = 1.15, 95% CI = [0.51–1.80], P = P < 0.001) and was more effective in younger patients (SMD = 1.43, 95% CI = [0.63–2.23], P = P < 0.001). Nursing interventions showed a statistically significant short-term effect on sexual satisfaction (SMD = 1.32, 95% CI = [0.44–2.20], P = 0.003) and a significant effect in older patients (SMD = 1.27, 95% CI = [0.46–2.08], P = 0.002).

**Conclusions:**

Nursing intervention may be an effective way to improve the sexual quality of life of patients with breast cancer. Nursing interventions had a stronger long-term effect on sexual function, and the group with the strongest effect is the younger patients. Nursing interventions showed a significant short-term effect on sexual satisfaction, and older patients had significant improvement in sexual satisfaction.

## 1. Introduction

According to the latest World Cancer Report 2020 by the International Agency For Research On Cancer of the World Health Organization [1], the number of new cases of breast cancer reached 2.26 million, exceeding lung cancer for the first time. Compared with other cancers, breast cancer has better prognosis, with a 5-year survival rate of more than 70% in most countries worldwide [2]. With the gradual improvement of survival rate, how to improve the quality of life in patients with breast cancer is becoming an increasingly important research topic.

Many treatments that patients with breast cancer undergo (e.g., radiotherapy and chemotherapy) can impair their sexual quality of life. Sexual health problems are common in patients with breast cancer, and between 23% and 85% of them may develop sexual problems [3]; the probability of sexual dysfunction is 74.4% [4]. In addition, sexual problems exist at all stages of breast cancer, affecting the sexual health of 60% of newly diagnosed, 64% of patients undergoing treatment, and 45% of patients completing treatment [5–7].

Sexual health is an integral part of the sexual quality of life of patients with breast cancer. A study found that oxytocin released during sexual activity can promote sleep [8]. Furthermore, sex can release endorphins, which prevent breast cancer progression by regulating stress and immune processes [9]. Another study suggested that increased serotonin during sexual contact can induce pleasurable emotions and reduce the risk of depression [10].

Medical and/or nursing interventions can be adopted for the sexual health of patients with breast cancer. Medical interventions include topical preparations, such as vaginal lubricants, as well as systemic drugs, such as androgens and antidepressants. Nursing interventions, including consultation, physical therapy, psychological therapy, and health education, are extensive. Taylor et al. [11] conducted a systematic review of sexual intervention in patients with breast cancer, whereas Seav et al. [12] conducted a systematic review of the management of sexual dysfunction in breast cancer survivors. However, neither study focused on the effectiveness of nursing interventions. A research [13] showed that 68% of patients with breast cancer hoped to obtain information related to breast cancer and sexual behavior. However, most patients are too ashamed to talk. Furthermore, the attitude of nurses in clinical sexual health care is negative due to time constraints, privacy considerations, and other obstacles.

This study systematically summarizes the effectiveness of nursing interventions on the sexual quality of life of patients with breast cancer, aiming to provide evidence-based evidence for relevant clinical nursing practice, raise nurses’ attention to sexual health care for patients with breast cancer, and ultimately improve the quality of life of patients.

## 2. Methods

This study was conducted in accordance with the Preferred Items for Systematic Review and Meta-analyses (PRISMA) guidelines [14]. This study was not registered nor did it follow a protocol.

### 2.1. Search strategy

Our literature retrieval period was from inception to October 7, 2021. Electronic databases included PubMed, Web of Science, the Cochrane Library, JBI database, CINAHL, Embase, Spring, CNKI (China), WanFang (China), SinoMed (China), and WeiPu (China). The search words used were a combination of medical subject headings and keywords, such as “breast cancer,” “breast carcinoma,” “breast tumor,” “breast tumour,” “breast neoplasm,” “breast sarcoma,” “sex,” “sexual,” “sexuality,” “effect,” “efficacy,” “random,” “intervention,” and “impact.” The detailed search strategies for English databases are shown in S1 Table.

### 2.2. Inclusion and exclusion criteria

Two researchers independently conducted the initial search and selected eligible studies on the basis of the following criteria: (1) population: adults (≥18 years) diagnosed with breast cancer; (2) intervention: nursing interventions concerning sexual health problems of patients with breast cancer, including psychological education, cognitive therapy, psychological counseling, exercise intervention, physical intervention, and so on; medical measures, such as drugs and laser, not included; (3) control: routine care or blank; (4) design: randomized controlled trial or quasi experiment study; (5) outcomes: sexual quality of life, including sexual function or sexual satisfaction; (6) language: English or Chinese. Exclusion criteria were as follows: (1) lack or inability to extract evaluation results of sexual quality in patients with breast cancer; (2) repeated articles published by the same research group; (3) poor quality of literature evaluation results; (4) unavailability of data for protocols, reports, and conference papers.

### 2.3. Study selection

Two researchers independently conducted literature screening, and any disagreement was solved by negotiating with a third researcher.

### 2.4. Data extraction

Two researchers independently screened the literature, and any disagreement was discussed with a third researcher. The following data were extracted from each study: author, publication year, country, study design, sample size, mean age of participant, evaluation time of intervention, measures of intervention/control group, primary outcomes (sexual function, sexual satisfaction), and secondary outcomes (body image, anxiety, depression, general quality of life). If only 95% confidence interval (CI) was reported, then it was converted to standard deviation in accordance with the following formula: 95% CI = X±Z_(α/2)_·S_X_.

### 2.5. Quality assessment of studies

The quality of included studies was independently assessed by two researchers. The Cochrane risk-of-bias tool [15] was used to assess RCTs. If ≥3 fields were considered high risk, the overall bias of this study was high. The Joanna Briggs Institute critical appraisal tool was used to assess quasi experimental studies. Studies were rated as high quality if ≥ 70% of the appraisal tool items received a point, moderate quality if ≥ 65%, and low quality if ≤ 55%. Any disagreement was resolved with a third researcher.

### 2.6. Data synthesis and analysis

RevMan 5.3 software was used to analyze the extracted data. Continuous outcomes were analyzed using standardized mean difference (SMD). Risk ratio was calculated for dichotomous outcomes. Point estimates, 95% CI, and, *P* were used to report outcomes. *P* < 0.05 was considered statistically significant. *I*^*2*^ test was used to determine whether heterogeneity existed between studies. *P* < 0.1 or *I*^*2*^≥50% was interpreted as high heterogeneity, and then the random effect model was used for analysis. Begg’s funnel plot was used to determine whether publication bias existed. Sensitivity analysis was performed by excluding each study in turn to test the robustness and reliability of the pooled results.

## 3. Results

### 3.1. Study selection

**Fig 1:**
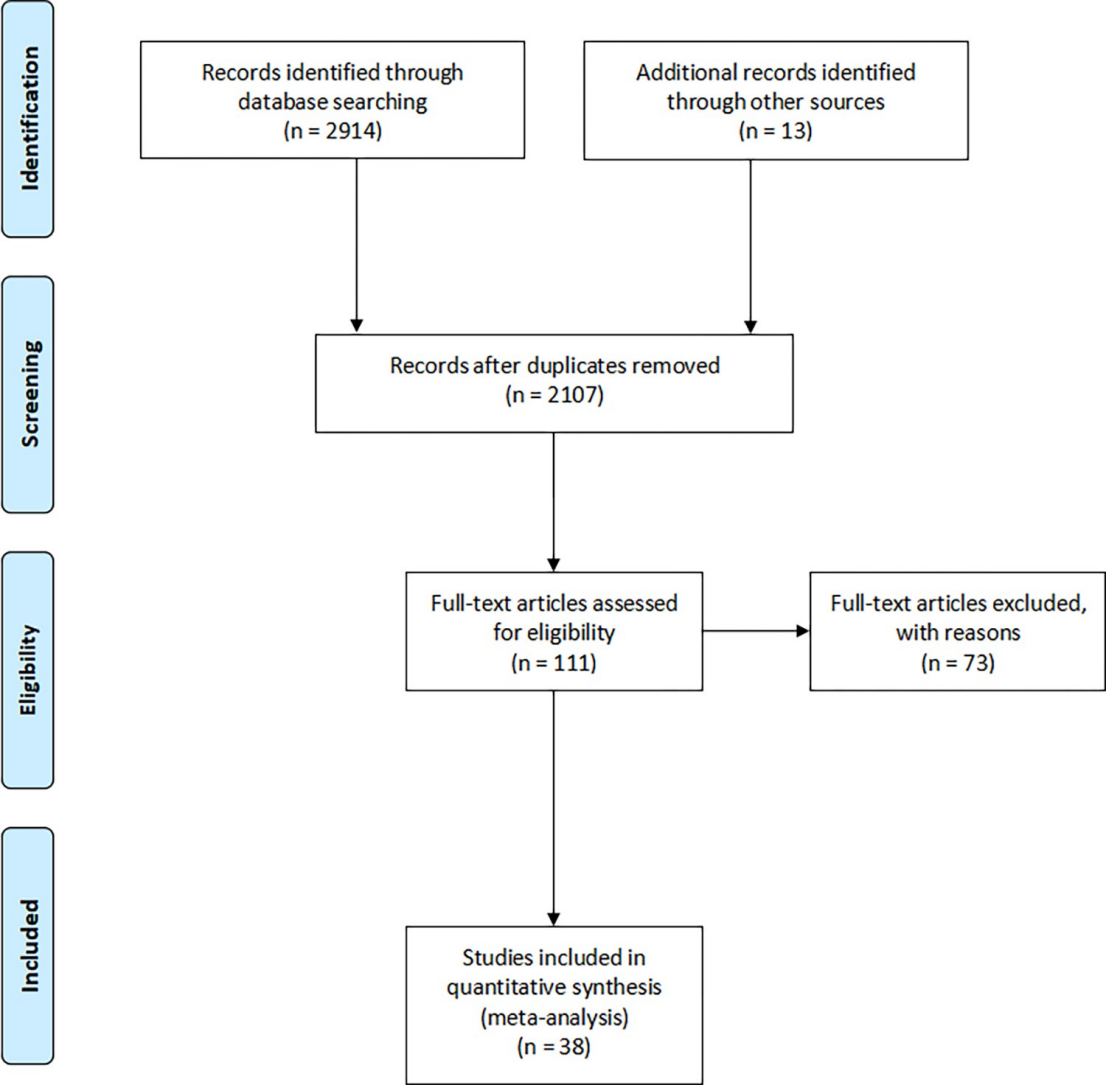
The PRISMA flow diagram.

A total of 2,914 articles were retrieved from electronic databases. 2,094 articles remained after removing duplicates. A total of 1,996 articles were excluded after reviewing the titles and abstracts. 98 articles underwent full text review, and 73 articles were excluded for the following reasons: not just nursing interventions (n = 3), without outcomes specified in the inclusion criteria (n = 5), not RCT or quasi experimental (n = 1), repeat published (n = 2), full text unavailable (n = 3), meeting abstract (n = 20), registration information or protocol (n = 26), unable to extract data (n = 12), and undesirable language (n = 1). A total of 13 articles were included by retrospecting the references. 38 studies were included in the quantitative synthesis (meta-analysis). The flow diagram of the selection procedure is shown in Fig 1.

### 3.2. Characteristics of included studies

This study involved 3,664 participants. About 38 studies were conducted in 11 countries: China (n = 13), America (n = 9), Iran (n = 7), the Netherlands (n = 2), Australia (n = 1), Brazil (n = 1), Finland (n = 1), Korea (n = 1), Canada (n = 1), Sweden (n = 1), and Greece (n = 1). The characteristics of included studies are presented in Table 1.

**Table 1:** Characteristics of included studies.

No	Author, year	Country	Study design	Sample size (Intervention/Control)	Mean age	Intervention	Control
1	Allen et al., 2002 [16]	America	RCT	76/73	42.3	A problem-solving therapy	Routine care
2	Kalaitzi et al., 2007 [17]	Greece	RCT	20/20	52.5	Psychosexual intervention	Routine care
3	Elkins et al., 2007 [18]	America	Quasi-experiment	16	53	Hypnosis	/
4	Wang et al., 2008 [19]	China	RCT	40/40	48	Psychological therapy	Routine care
5	Christensen et al., 2008 [20]	America	RCT	10/10	39.7	Couple counselling	Routine care
6	Salonen et al., 2009 [21]	Finland	Quasi-experiment	120/108	56.5	Telephone intervention	Routine care
7	Rowland et al., 2009 [22]	America	RCT	57/98	54.8	Psycho-educational group intervention	Routine care
8	Baucom et al., 2009 [23]	America	RCT	8/6	50	A couple-based intervention	Routine care
9	Chen et al., 2011 [24]	China	RCT	40/40	42.8	Psychological, behaviour therapy	Routine care
10	Jun et al., 2011 [25]	Korea	RCT	22/23	46	Psychological therapy, relationship intervention	Routine care
11	Duijts et al., 2012 [26]	Netherland	RCT	22/65	48.4	Cognitive behavioural therapy, physical exercise	Routine care
				35/65	48		
				26/65	47.7		
12	Wang et al., 2013 [27]	China	RCT	40/40	38.5	Psychological therapy	Routine care
13	Björneklett et al., 2013 [28]	Sweden	RCT	136/125	58.6	Support group intervention	Routine care
14	Kashani et al., 2014 [29]	America	RCT	12/12	47.9	Support group intervention	Routine care
15	Li et al., 2014 [30]	China	RCT	30/30	44	Continuous nursing, routine care	Routine care
16	Anderson et al., 2015 [31]	Australia	RCT	26/25	49.2	A multimodal lifestyle program	Routine care
17	Pan et al., 2016 [32]	China	RCT	80/80	36.1	Case work	Routine care
18	Hummel et al., 2017 [33]	Netherland	RCT	84/85	51.1	Internet-based cognitive behavioural therapy	Routine care
19	Shayan et al., 2017 [34]	Iran	Quasi-experiment	52/52	48.4	Stress management	Routine care
20	Esplen et al., 2018 [35]	Canada	RCT	131/63	49.8	Group psychosocial intervention	Routine care
21	Pan et al., 2018 [36]	China	RCT	49/49	46.4	Family support, cognitive behavioural therapy	Routine care
22	Li et al., 2018 [37]	China	RCT	100/103	/	Sex education curriculum	Routine care
23	Jalambadani et al., 2018 [38]	Iran	Quasi-experiment	60/60	46.1	Education based on theory of planned behaviour	Routine care
24	Peng et al., 2019 [39]	China	RCT	10/50	41.5	Sexual health education, routine care	Routine care
25	Wang et al., 2019 [40]	China	RCT	50/50	38.2	Couple-centered nursing intervention, routine care	Routine care
26	Zhang et al., 2019 [41]	China	RCT	44/44	46.3	Continuous nursing, routine care	Routine care
27	Reese et al., 2019 [42]	America	RCT	19/9	54.1	Couple-based intervention addressing sexual concerns	Routine care
28	Fatehi et al., 2019 [43]	Iran	RCT	51/49	44.3	Psychosexual counselling	Routine care
29	de Almeida et al., 2020 [44]	Brazil	Quasi-experiment	10/8	54.6	PLISSIT model intervention	Routine care
30	Bober et al., 2020 [45]	America	Quasi-experiment	19	38.6	Psychosexual intervention	Routine care
31	Abedini et al., 2020 [46]	Iran	RCT	40/40	43.5	Psychoeducation intervention	Routine care
32	Khoei et al., 2020 [47]	Iran	RCT	22/18	39.4	PLISSIT-based counselling	Routine care
				25/18	40.7		
33	Zhou et al., 2020 [48]	China	RCT	80/80	35.7	Recreational nursing intervention based on "family approval"	Routine care
34	Bagherzadeh et al., 2020 [49]	Iran	RCT	22/24	46.8	Mindfulness-based stress reduction training	Routine care
35	Esmkhani et al., 2021 [50]	Iran	RCT	22/18	39.4	Individual counselling based on the PLISSIT model	Routine care
				25/18	40.7		
36	Wang et al., 2021 [51]	China	RCT	50/50	37.7	Psychosexual intervention	Routine care
37	Guo et al., 2021 [52]	China	RCT	48/48	42.8	Family support care, routine care	Routine care
38	Reese et al., 2021 [53]	America	RCT	71/69	56	Multimedia intervention	Routine care

### 3.3. Risk of bias of included studies

**Fig 2:**
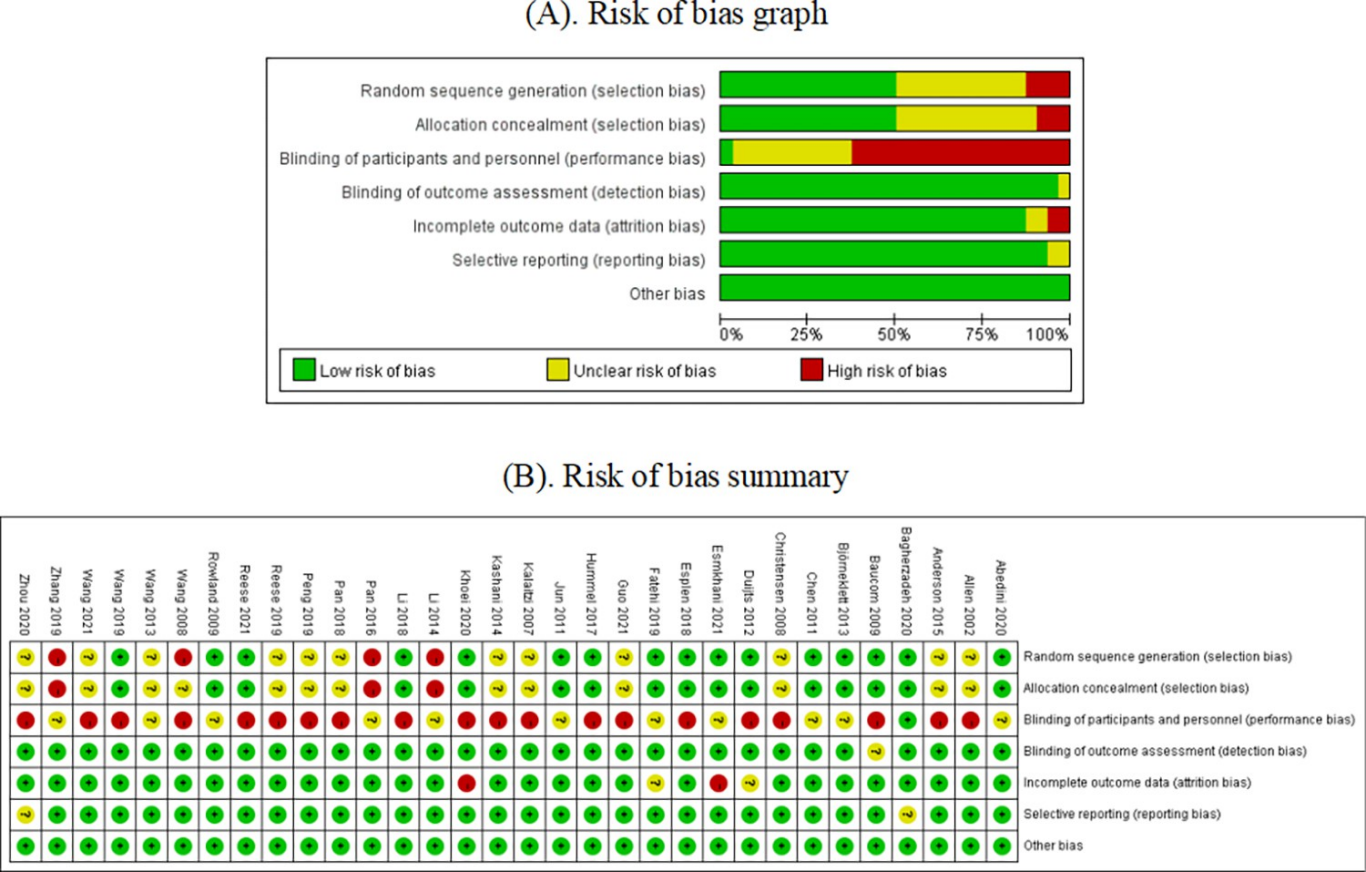
Quality assessment of included RCTs.

**Fig 3:**
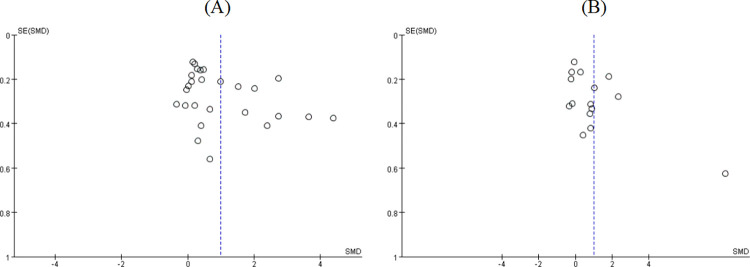
Funnel plots for (A) sexual function; (B) sexual satisfaction.

The quality assessment of included RCTs is shown in Fig 2. About 17 (51.5%) studies used random assignment and allocation concealment. These studies were unable to blind participants and interveners due to the specificity of intervention. Thus, 21 (63.6%) studies were assessed as high risk in the “performance bias” item. The quality assessment of quasi experimental studies is shown in Table 2. Two studies reached “not applicable” in baseline comparison and similarity in intervention of interest. The remaining two studies reached “no reported” due to loss of follow-up. The rest of the evaluations were "Yes." Funnel plots were made for groups including more than 10 studies; “sexual function” did not have publication bias, whereas publication bias may exist in “sexual satisfaction” (Fig 3). The evaluation results of 38 studies met the inclusion criteria.

**Table 2:** Quality appraisal of quasi-experimental studies (N = 6).

Questions	Shayan et al., 2017 [34]	Jalambadani et al., 2018 [38]	de Almeida et al., 2020 [44]	Bober et al., 2020 [45]	Salonen et al., 2009 [21]	Elkins et al., 2007 [18]
Is it clear in the study what is the cause and what is the effect? (i.e. there is no confusion about which variable comes first?)	√	√	√	√	√	√
Were the participants included in any comparisons similar?	√	√	√	NA	√	NA
Were the participants included in any comparisons receiving similar treatment/care, other than the exposure or intervention of interest?	√	√	√	NA	√	NA
Was there a control group?	√	√	√	√	√	√
Were there multiple measurements of the outcomes both pre and post the intervention/exposure?	√	√	√	√	√	√
Was follow up complete and if not, were differences between groups in terms of their follow up adequately described and analyzed?	NR	NR	√	√	√	√
Were the outcomes of participants included in any comparisons measured in the same way?	√	√	√	√	√	√
Were outcomes measured in a reliable way?	√	√	√	√	√	√
Was appropriate statistical analysis used	√	√	√	√	√	√
Overall (maximum 9; minimum: 1)	8	8	9	7	9	7

Note. √ = Yes, × = no, NA = not applicable, NR = not reported.

### 3.4. Primary outcomes (sexual quality of life)

#### 3.4.1. Sexual function

**Fig 4:**
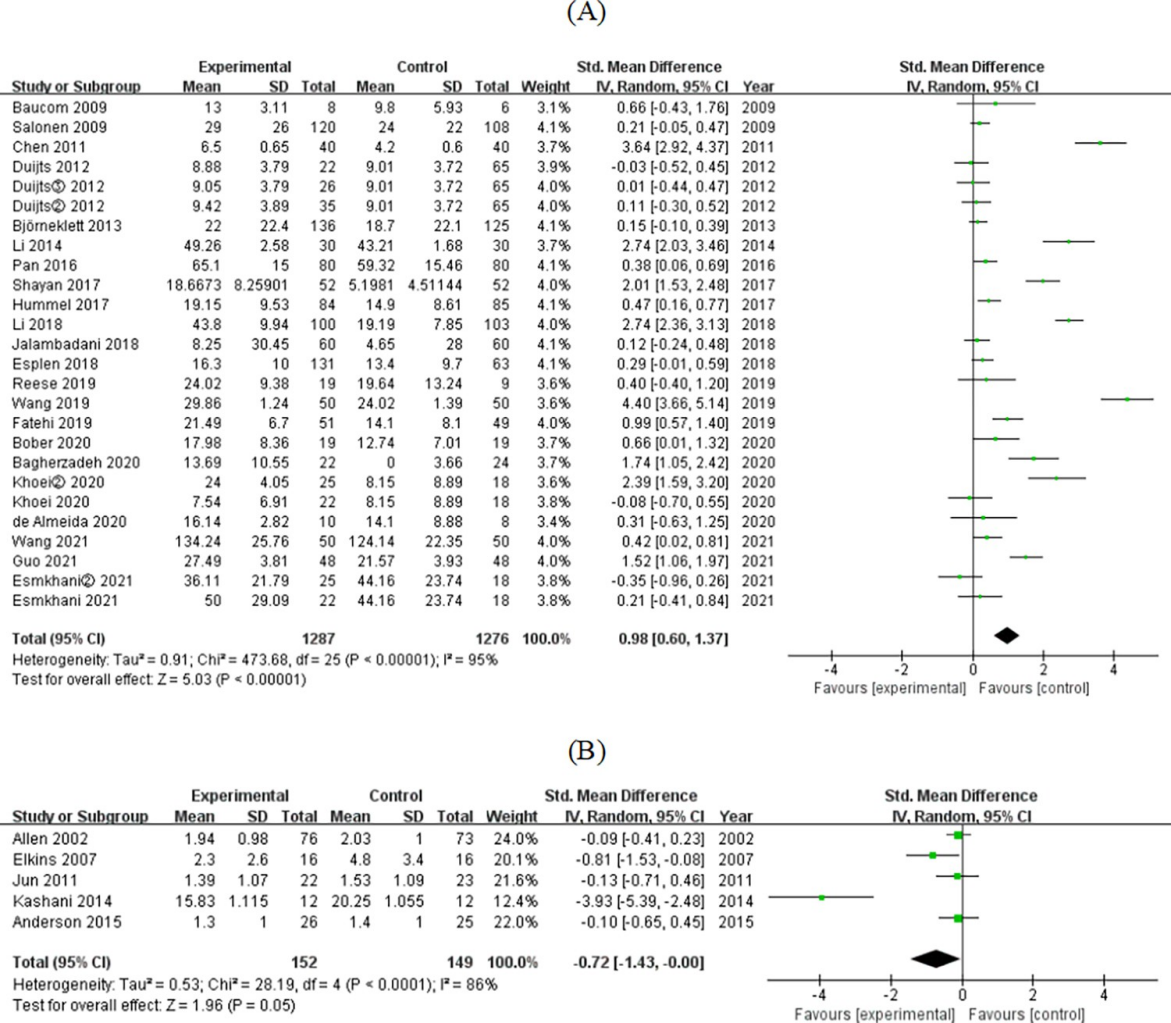
Forest plot of effect of nursing interventions on sexual function in studies with (A) high-priority scale; (B) low-priority scale.

Sexual function was examined in 27 studies. In the scales used in these studies, some scales represented better outcomes with higher scores (high-priority scale), whereas others represented better outcomes with lower scores (low-priority scale). The effect values were combined separately. In 22 studies with high-priority scale, 26 sets of data were extracted. The results showed that participants in the nursing intervention group had a significantly higher sexual function than the control group (SMD = 0.98, 95% CI = [0.60–1.37], *P* < 0.001) (Fig 4A). In five studies with low-priority scale, the results showed that the sexual function of the participants in the nursing intervention group was better than that in the control group, and the differences were very close to statistical significance (SMD = −0.72, 95% CI = [−1.43–−0.00], *P* = 0.05) (Fig 4B).

#### 3.4.2. Sexual satisfaction

A total of 14 studies reported sexual satisfaction in the form of quantitative data, from which 15 sets of data involving a total of 1,340 participants, were extracted. Results displayed that the sexual satisfaction of the nursing intervention group was significantly higher than that of the control group (SMD = 0.99, 95% CI = [0.41–1.57], *P* < 0.001) (S1A Fig). Two other studies reported qualitative data on sexual satisfaction, and the results were equally statistically significant (OR = 19.22, 95% CI = [2.42–152.72], *P* = 0.005) (S1B Fig). A study [29] reported quantitative satisfaction results, but it used low-priority scale and thus could not be included in data combination. Although the effect was not statistically significant, sexual satisfaction in the nursing intervention group was stronger than that in the control group (MD = −0.78, 95% CI = [−1.61–0.06], *P* = 0.510).

### 3.5. Secondary outcomes

#### 3.5.1. Body image

The scales used to measure body image included high-priority scale and low-priority scale. In the high-priority scale group, the analysis of seven sets of data from six studies showed that body image in the nursing intervention group was higher than that that in the control group, but no statistical differences were found (SMD = 0.17, 95% CI = [−0.08–0.41], *P* = 0.19) (S2A Fig). In the low-priority scale group, synthetic data suggested that body image had a slight difference between two groups (SMD = −0.79, 95% CI = [−1.66–0.09], *P* = 0.08) (S2B Fig).

#### 3.5.2. Psychological outcomes

Eight studies, including a total of 597 participants, reported anxiety. The results showed that nursing interventions had minimal effects of improving participants’ anxiety (SMD = −0.45, 95% CI = [−0.93–0.02], *P* = 0.06) (S2C Fig).

Eight studies, involving 657 participants, reported depression. The results showed that depression of participants was significantly improved (SMD = −1.16, 95% CI = [−2.08–−0.24], *P* = 0.01) (S2D Fig).

#### 3.5.3. General quality of life

Nine sets of data were extracted from eight studies, involving 973 participants. Data consolidation revealed that nursing interventions significantly improved participants’ general quality of life (SMD = 0.20, 95% CI = [0.08–0.33], *P* = 0.002) (S2E Fig).

### 3.6. Subgroup analysis

#### 3.6.1. Subgroup analysis based on the evaluation time of intervention effect

The results of sexual function were subgroup analyzed in accordance with the different evaluation times of nursing intervention effect in studies with high-priority scale (S3A Fig). The results showed that nursing interventions could not only significantly improve the sexual function of the participants in short term (<3 months) (SMD = 0.81, 95% CI = [0.37–1.24], *P* < 0.001) but also had a notable long-term effect (≥3 months) (SMD = 1.15, 95% CI = [0.51–1.80], *P* < 0.001).

We also performed a subgroup analysis of evaluation time for sexual satisfaction in studies reporting quantitative results (S3B Fig). The results showed that nursing interventions had a statistically significant short-term effect (<3 months) on participants’ sexual satisfaction (SMD = 1.32, 95% CI = [0.44–2.20], *P* = 0.003) but not a long-term effect (≥3 months) (SMD = 0.23, 95% CI = [−0.22–0.69], *P* = 0.32).

#### 3.6.2. Subgroup analysis based on age of participants

Subgroup analysis based on age in studies with high-priority scale (one study [37] was unable to obtain age data) (S3C Fig) showed that nursing interventions significantly improved sexual function in younger participants (≤44 years old) (SMD = 1.43, 95% CI = [0.63–2.23], *P* < 0.001), as well as older participants (>44 years old) (SMD = 0.50, 95% CI = [0.22–0.79], *P* < 0.001). The effect of nursing interventions on younger participants was stronger than that on older participants.

Subgroup analysis based on age in studies that reported quantitative results (S3D Fig) showed that nursing interventions significantly improved sexual satisfaction among older participants (>44 years old) (SMD = 1.27, 95% CI = [0.46–2.08], *P* = 0.002), but for younger patients (≤44 years old), although improvement was noted, no statistical differences were observed (SMD = 0.61, 95% CI = [−0.13–1.35], *P* = 0.11).

## 4. Discussion

### 4.1. Effectiveness of nursing interventions

A total of 38 studies were included in this systematic review and meta-analysis. Pooled results showed that nursing interventions significantly improved participants’ sexual quality of life, including sexual function and sexual satisfaction, compared with routine care. In addition, nursing interventions improved participants’ depression and general quality of life. However, the improvement on body image and anxiety were nonsignificant.

Nursing interventions in this review mainly included psychological intervention, educational intervention, and physical intervention. Psychological intervention, such as psychosexual counseling [43] and Internet-based counseling cognitive behavioral therapy [33], was the most frequently used intervention. Some studies used educational intervention, such as sex education curriculum [37] and sexual health education [39]. Physical intervention, such as physical exercise [26] and a multimodal lifestyle program [31], was also used. Many studies combined nursing interventions with emerging Internet technology. For example, Hummel et al. used Internet-based cognitive behavioral therapy [33], and Anderson et al. used multimedia technology [31]. At present, various kinds of nursing interventions are implemented for the sexual health of patients with breast cancer. More high-quality evidence is needed to further prove the effectiveness and feasibility of specific types of nursing interventions.

Our study showed that nursing interventions had a positive effect on the sexual quality of life of patients with breast cancer. Previous studies have demonstrated the effectiveness of nursing interventions in improving sexual function in primiparas [54] and psychopaths [55]; this finding is consistent with our study. In addition, nursing interventions significantly improved participants’ sexual satisfaction. Overall, before implementing nursing interventions, many participants ignored their normal sexual needs and turned to more urgent treatment needs [56]. Through nursing intervention, participants’ erroneous cognitions might be changed; thus, high sexual satisfaction could be achieved.

In addition, no statistical differences were found in the effect of nursing interventions on participants’ body image in either high-priority or low-priority scale groups. This result might be due to the low number of included studies. Another possible reason is that in some of the included studies, the primary goal of nursing interventions was to improve sexual quality; thus, the changes in body image were not significant. Concluding that nursing interventions have no effect on body image may be premature. Nursing interventions improved body image scores without statistical differences. In terms of mental health, meta-analysis indicated that nursing interventions had a nonsignificant effect on participants’ anxiety but could significantly improve depression. This result may be related to the few participants in the anxiety group. Similarly, concluding that nursing interventions have no effect on anxiety may be premature because anxiety scores were reduced in the nursing intervention group, even if it was nonsignificant. A study [57] suggested that nursing interventions improved the general quality of life of participants with statistical significance; this finding is proven in our study.

A research [58] investigated the information sources of patients obtaining sexual health knowledge, and 66.4% of patients with breast cancer preferred to obtain relevant knowledge from nurses, followed by doctors. Most sexual problems in patients with breast cancer were not organic but mainly caused by the psychological and cognitive factors of patients and their spouses [59–61]; this finding further highlights the necessity of nursing interventions in patients’ sexual health. Compared with doctors, nurses have more contact with patients. In view of the current situation that most patients with breast cancer need sexual knowledge but do not often bring this up initiatively, nurses are more suitable candidates to deal with the sexual problems of patients.

Sexual discussion between nurses and patients are hindered by several factors. First, some patients are reluctant to ask health care providers for sexual health information because of traditional beliefs. A study [62] revealed that only 30% of breast cancer couples discussed sexual issues with medical personnel. In addition to the patient’s subjective reasons, nurses also need to take some responsibility. The reasons conspiring to the neglect of nurses in sexual health care are plentiful; some are subjective reasons, such as negative attitude of nurses toward sexual health care [63], opinion of nurses that sexual issues are not the main concern of patients [64, 65], and limited knowledge about sexual health [66]. Nevertheless, other reasons, such as lack of time [67], constraints of traditional culture, and fear of invading patients’ privacy [66], are objective. One of the current priorities is to promote nurses’ awareness of and professionalism in terms of sexual health care. Thus, nurses must be provided with relevant training to improve their attitudes and skills. Changes in systems and policies to increase nurses’ time spent on sexual health care are also needed.

### 4.2. Subgroup analysis

A subgroup analysis was performed on the basis of the effect evaluation time to analyze the continuity of the effect of nursing interventions on sexual quality of life. For sexual function, subgroup analysis showed that the effect of nursing interventions was short term and long term, and the latter was superior to the former. This result indicated the stability of nursing interventions to improve participants’ sexual function. For sexual satisfaction, subgroup analysis showed that nursing interventions can improve the sexual satisfaction of patients with breast cancer in the short term; however, no long-term stability is expected. This result might be related to the lack of continuity of care. Sexual satisfaction is a subjective indicator, and if participants do not receive consistent care, their ratings may decline over time. However, due to the lack of data on intervention time in most studies, there was no subgroup analysis on it, otherwise, the results of subgroup analysis on effect evaluation time could be supplemented, and conclusions can be refined.

In addition, a subgroup analysis was performed contraposing the age of the participants. For sexual function, subgroup analysis showed that sexual function in younger and older patients could be significantly improved by nursing interventions, and the effectiveness is better in younger patients than in older ones. In clinical nursing, substantial attention should be paid to the sexual health of younger patients; moreover, initiatives must be taken to find problems and provide solutions, which may lead to strong feedback. For sexual satisfaction, subgroup analysis showed that nursing interventions could significantly improve sexual satisfaction in older participants but not in younger participants. The reason might be that older patients were more likely to ignore sexual health needs and feelings prior to nursing interventions [68]; lower baseline levels led to more significant improvements in sexual satisfaction. Younger patients possibly have higher requirements for sexual life; thus, achieving a marked improvement is difficult for them. This finding may also be related to the smaller sample size of the younger group.

### 4.3. Implications for nursing practice and further research

Nurses can improve the sexual problems of patients with breast cancer by providing timely, targeted sexual health care. As early as 1974, the American Nurses Association argued that sexual health care was an essential part of nursing [69]. “Sexuality” also appeared as a separate category of nursing diagnosis in the nursing diagnosis developed by the North American Nursing Diagnostic Association. At present, including sexual health care as a standard and routine of care in nurses’ to-do lists should be considered. In the practical work of sexual health care, nurses should enhance continuity of care and pay more attention to young patients. Furthermore, the significance of sexual health in clinical nursing must be gradually instilled, nurses’ subjective attention to sexual health care must be improved, nurse-led sexual health care must be refined and improved, and then promote patients’ awareness of sexual health. Eventually, the sexual quality of life and survival quality in patients with breast cancer can be improved.

### 4.4. Limitations

This review was confined to breast cancer. Perhaps, additional data could be provided by including studies involving general sexual dysfunction patients. In addition, race, job, and other data of participants were not limited and thus might lead to the heterogeneity of this study. Although sensitivity analysis showed robust results, the publication bias of “sexual satisfaction” was relatively serious. The included studies incorporated quasi experimental studies, which perhaps drew biased conclusions. In addition, differences in measurement tools also resulted in a decrease in the number of studies in some meta-analysis groups. In some subgroup analyses, negative results were obtained, which might be due to small sample size, thus, opportunities to identify differences between the intervention and control groups perhaps be missed. Despite the use of a comprehensive search strategy, the full text of some studies was still unavailable, and gray literature could possibly be ignored.

## 5. Conclusions

Nursing interventions significantly improved the sexual quality of life of patients with breast cancer, including sexual function and sexual satisfaction. It also significantly improved their depression and general quality of life but did not significantly improve body image or anxiety. The long-term effect of nursing interventions on the sexual function of patients with breast cancer is stronger, and younger patients benefited most. Nursing interventions had a significant short-term effect on sexual satisfaction, and it significantly increased sexual satisfaction in older patients. This study points out that continuing nursing care must be strengthened, focusing on younger patients with targeted measures for their needs in sexual health. More well-designed RCTs are needed to confirm the optimal duration and type of nursing interventions in sexual health care. Professional training in sexual health for nurses should also be on the agenda.

## Supporting information

S1 Checklist PRISMA Checklist. (DOC)

S1 Table Search strategies for each database. (DOC)

S2 Table List of excluded studies. (DOCX)

S3 Table Detailed data. (XLSX)

S1 Fig Forest plot of the effect of nursing interventions on sexual satisfaction. (TIF)

S2 Fig Forest plot of the effect of nursing interventions on secondary outcomes. (TIF)

S3 Fig Forest plot of subgroup analysis. (TIF)
