# Supplementary material for: Effectiveness of nursing interventions on the sexual quality of life of patients with breast cancer: A systematic review and meta-analysis
Source: PLoS One. 2022 Nov 3;17(11):e0277221. doi: 10.1371/journal.pone.0277221 (PMC9632802; doi:10.1371/journal.pone.0277221)
Supplement: S1 Table — (DOC) [file pone.0277221.s002.doc]

**S1 Table. Search strategies for each database**

| **PubMed**  ((breast cancer*[Title] OR breast carcinoma*[Title] OR breast tumor*[Title] OR breast tumour*[Title] OR breast neoplasm*[Title] OR breast sarcoma*[Title]) AND (sex*[Title/Abstract] OR sexual[Title/Abstract] OR sexuality[Title/Abstract])) AND (effect*[Title] OR efficacy[Title] OR random*[Title] OR intervention*[Title] OR impact*[Title])  **Web of Science**  TI=(breast cancer* OR breast carcinoma* OR breast tumor* OR breast tumour* OR breast neoplasm* OR breast sarcoma*) AND TS=(sex* OR sexual OR sexuality) AND TI=(effect* OR efficacy OR random* OR intervention* OR impact*)  **the Cochrane Library**  (breast cancer* OR breast carcinoma* OR breast tumor* OR breast tumour* OR breast neoplasm* OR breast sarcoma*)[Record Title] AND (sex* OR sexual OR sexuality)[Title Abstract Keyword] AND (effect* OR efficacy OR random* OR intervention* OR impact*)[Record Title]    **JBI database**  Title=(breast cancer* OR breast carcinoma* OR breast tumor* OR breast tumour* OR breast neoplasm* OR breast sarcoma*) AND Abstract=(sex* OR sexual OR sexuality) AND Title=(effect* OR efficacy OR random* OR intervention* OR impact*)  **CINAHL**  TI ( (breast cancer* OR breast carcinoma* OR breast tumor* OR breast tumour* OR breast neoplasm* OR breast sarcoma* ) AND AB ( sex* OR sexual OR sexuality ) AND TI ( effect* OR efficacy OR random* OR intervention* OR impact* )  **Embase**  ('breast cancer*':ti OR 'breast carcinoma*':ti OR 'breast tumor*':ti OR 'breast tumour*':ti OR 'breast neoplasm*':ti OR 'breast sarcoma*':ti) AND (sex*:ti OR sexual:ti OR sexuality:ti) AND (effect*:ti OR efficacy:ti OR random*:ti OR intervention*:ti OR impact*:ti)  **Spring**  (breast AND cancer* AND OR AND breast AND carcinoma* AND OR AND breast AND tumor* AND OR AND breast AND tumour* AND OR AND breast AND neoplasm* AND OR AND breast AND sarcoma*) AND AND AND (sex* AND OR AND sexual AND OR AND sexuality) AND AND AND (effect* AND OR AND efficacy AND OR AND random* AND OR AND intervention* AND OR AND impact*) |
| --- |
