# Supplementary material for: Effectiveness of nursing interventions on the sexual quality of life of patients with breast cancer: A systematic review and meta-analysis
Source: PLoS One. 2022 Nov 3;17(11):e0277221. doi: 10.1371/journal.pone.0277221 (PMC9632802; doi:10.1371/journal.pone.0277221)
Supplement: S2 Table — (DOCX) [file pone.0277221.s003.docx]

**S2 Table. List of excluded studies**

| **Reasons for exclusion** | **Number of studies** | **List of excluded articles** |  |
| --- | --- | --- | --- |
| Not just nursing interventions | 3 | Juraskova I, Jarvis S, Mok K, Peate M, Meiser B, Cheah BC, et al. The acceptability, feasibility, and efficacy (phase I/II study) of the OVERcome (Olive Oil, Vaginal Exercise, and MoisturizeR) intervention to improve dyspareunia and alleviate sexual problems in women with breast cancer. J Sex Med. 2013;10(10):2549-58. http://doi.org/10.1111/jsm.12156 | |
|  |  | Advani P, Brewster A, Baum G, Schover L, Brewster AM, Baum GP, et al. A pilot randomized trial to prevent sexual dysfunction in postmenopausal breast cancer survivors starting adjuvant aromatase inhibitor therapy. J Cancer Surviv. 2017;11(4):477-85. http://doi.org/10.1007/s11764-017-0606-3 | |
|  |  | Scott JL, Halford WK, Ward BG. United we stand? The effects of a couple-coping intervention on adjustment to early stage breast or gynecological cancer. J Consult Clin Psychol. 2004;72(6):1122-35. http://doi.org/10.1037/0022-006X.72.6.1122 | |
| Without outcomes specified in the inclusion critieria | 5 | Wang WL, Wang LC, Yang E, Wen ZH. Analysis of influencing factors of psychosexual disorder in patients with breast cancer and nursing countermeasures. J Adv Health. 2016;23:25. | |
|  |  | Price-Blackshear MA, Pratscher SD, Oyler DL, Armer JM, Cheng A, Cheng MX, et al. Online couples mindfulness-based intervention for young breast cancer survivors and their partners: A randomized-control trial. J Psychosoc Oncol. 2020;38(5):592-611. http://doi.org/10.1080/07347332.2020.1778150 | |
|  |  | Katz A. Sexually Speaking. Breast Cancer and Women's Sexuality: Acknowledging and discussing the consequences of treatment. AJN American Journal of Nursing. 2011;111(4):63-6. http://doi.org/10.1097/01.NAJ.0000396560.09620.19 | |
|  |  | Farnam F, Khakbazan Z, Barjasteh S, Nedjat S, Razavi Dizaji S. The Effect of Good Enough Sex (GES) Model-Based Sexual Counseling Intervention on the Body Image in Women Surviving Breast Cancer: A Randomized Clinical Trial. Asian Pacific journal of cancer prevention : APJCP. 2021;22(7):2303-10. http://doi.org/10.31557/APJCP.2021.22.7.2303 | |
|  |  | Cullen K, Fergus K. Acceptability of an online relational intimacy and sexual enhancement (iRISE) intervention after breast cancer. Journal of Marital & Family Therapy. 2021;47(2):515-32. http://doi.org/10.1111/jmft.12516 | |
| Not RCT or quasi-experimental | 1 | Liu Q, Zhang CP, Kuang LY, Wang MG. Application of sexual rehabilitation guidance in female breast cancer after surgery. J Qilu Nurs. 2008;14(10):91-2. http://doi.org/CNKI:SUN:QLHL.0.2008-20-078 | |
| Repeat published | 2 | Hummel SB, van Lankveld JJDM, Oldenburg HSA, Hahn DEE, Kieffer JM, Gerritsma MA, et al. Sexual Functioning and Relationship Satisfaction of Partners of Breast Cancer Survivors Who Receive Internet-Based Sex Therapy. J Sex Marital Ther. 2019;45(2):91-102. http://doi.org/10.1080/0092623X.2018.1488325 | |
|  |  | Hummel SB, van Lankveld JJDM, Oldenburg HSA, Hahn DEE, Kieffer JM, Gerritsma MA, et al. Internet-Based Cognitive Behavioral Therapy Realizes Long-Term Improvement in the Sexual Functioning and Body Image of Breast Cancer Survivors. J Sex Marital Ther. 2018;44(5):485-96. http://doi.org/10.1080/0092623X.2017.1408047 | |
| Full text unavailable | 3 | Zimmermann T, Heinrichs N. Effects of a psychosocial intervention for couples on sexuality when the woman has breast cancer. Z Gesundheitspsychol. 2011;19(1):23-34. http://doi.org/https://dx.doi.org/10.1026/0943-8149/a000037 | |
|  |  | WANG J. Effect of cooperative nursing intervention on sexual rehabilitation and negative emotions of patients after modified radical mastectomy for breast cancer. Chinese Nursing Research. 2020(19):3523-6. http://doi.org/10.12102/j.issn.1009-6493.2020.19.034 | |
|  |  | Reese JB, Lepore SJ, Daly MB, Handorf E, Sorice KA, Porter LS, et al. Can a brief multimedia intervention facilitate breast cancer patients' communication about sexual health? Findings from a randomized controlled trial. J Clin Oncol. 2020;38S(15). | |
| Meeting abstract | 20 | Stark SS, Kwan B, Myers E, Natarajan L, Su H. Randomized controlled trial of the effect of a reproductive health survivorship care plan on fertility and pregnancy concerns, vasomotor symptoms, sexual health, and contraception in young breast cancer survivors. 2018;110(4, Suppl.):e48. http://doi.org/10.1016/j.fertnstert.2018.07.149 | |
|  |  | Qiu J. Effectiveness of sexual educational program on quality of life of breast cancer patients. Cancer Nurs. 2017;40(6 Supplement 1):E26. http://doi.org/10.1097/NCC.0000000000000555 | |
|  |  | Porter-Steele J, Anderson D, McGuire A, Seib C. Utilising a nurse led holistic lifestyle intervention in reducing sexuality concerns in women after breast cancer - The Pink Women's Wellness Program results. Eur J Cancer. 2016;572:S25. | |
|  |  | Paterson C, Lengacher C. STATE OF THE SCIENCE: RANDOMIZED CONTROLLED TRIALS IMPLEMENTING COUPLES INTERVENTIONS ADDRESSING SEXUALITY AFTER BREAST CANCER TREATMENT. Oncol Nurs Forum. 2014;41(2):E124. | |
|  |  | Paterson C, Lengacher C. SEXUAL DISTRESS, BODY IMAGE AND MBSR IN YOUNGER BREAST CANCER SURVIVORS: DESIGN OF A DOCTORAL DISSERTATION WITHIN THE CURRENT R01 MBSR SYMPTOM CLUSTER TRIAL FOR BREAST CANCER SURVIVORS RANDOMIZED CONTROLLED TRIAL. Oncol Nurs Forum. 2013;40(3):E300-1. | |
|  |  | Paterson C, Lengacher C, Donovan K, Kip K, Tofthagen C. The effects of MBSR (BC) on sexual distress and body image disturbance in breast cancer survivors. 2015;24:287. http://doi.org/10.1002/pon.3874 | |
|  |  | Park H, Kang EY, Kim J, Kim S, Kim J, Choi S. Effect of beauty treatment on the body image, sexual satisfaction, and coping in patients with breast cancer. Psycho-Oncology. 2014;23(SUPPL. 3):200. http://doi.org/10.1111/j.1099-1611.2014.3695 | |
|  |  | Juraskova I, Jarvis S, Mireskandari S, Mok K, Peate M, Meiser B, et al. An intervention to help women 'OVERcome' dyspareunia and sexual problems after breast cancer treatment. Psycho-Oncology. 2011;20(SUPPL. 2):274-5. http://doi.org/10.1002/pon.2078 | |
|  |  | Juraskova I, Jarvis S, Peate M, Meiser B, Mireskandari S, Mok K, et al. Helping women "overcome" sexual problems after breast cancer treatment: An intervention study. Support Care Cancer. 2011;19(2 SUPPL. 1):S331. http://doi.org/10.1007/s00520-011-1184-y | |
|  |  | Juraskova I, Jarvis S, Mok K, Peate M, Meiser B, Mireskandari S, et al. OVERcome: An intervention study to improve sexual function in women with breast cancer. J Clin Oncol. 2011;29(15 SUPPL. 1). | |
|  |  | Hummel S, Van Lankveld J, Oldenburg H, Hahn D, Broomans E, Aaronson N. Efficacy of internet-based cognitive behavioural therapy in improving sexual functioning of breast cancer survivors with a DSM-IV diagnosis of sexual dysfunction: results of a multicenter, randomized controlled trial. 2016;25:54-5. http://doi.org/10.1002/pon.4272 | |
|  |  | Hummel L, Van Lankveld J, Oldenburg H, Hahn D, Kieffer J, Gerritsma M, et al. 101 - Efficacy of internet-based cognitive behavioral therapy in improving sexual functioning of breast cancer survivors with a DSM-IV diagnosis of sexual dysfunction: results of a randomized controlled trial...ECCO: 2017 European Cancer Congress 27 January 2017 - 30 January 2017. Eur J Cancer. 2017;72:S7-8. http://doi.org/10.1016/S0959-8049(17)30105-3 | |
|  |  | Hamzehgardeshi Z, Rezaei M, Elyasi F, Janbabai G, Moosazadeh M, Dayhimi M. The effect of supportive program on sexual satisfaction in patients with breast cancer. 2018;143:453. http://doi.org/10.1002/ijgo.12582 | |
|  |  | Esplen MJ, Clarke S, Fergus K, Warner E, Wong J. Body image, sexual and psychosocial functioning in women with breast cancer: Can we fix what we've broken? a randomized trial. Psycho-Oncology. 2009;18(SUPPL. 2):S73. http://doi.org/10.1002/pon.1594 | |
|  |  | Hummel L, Van Lankveld J, Oldenburg H, Hahn D, Kieffer J, Gerritsma M, et al. Efficacy of internet-based cognitive behavioral therapy in improving sexual functioning of breast cancer survivors with a DSM-IV diagnosis of sexual dysfunction: results of a randomized controlled trial. 2017;72:S7-8. | |
|  |  | Esplen MJ, Wong J, Warner E, Toner B. Can we fix what we've broken? Randomized controlled trial of a group therapy to address body image disturbance and sexuality following treatment with breast cancer. 2014;23:85. http://doi.org/10.1111/j.1099-1611.2014.3694 | |
|  |  | Behzadi Pour S, Naziri G. The efficacy of cognitive behavior therapy on sexual satisfaction in women with breast cancer. 2015;12:368. | |
|  |  | Behzadi Pour S, Naziri G. Effectiveness of cognitive behavior therapy on sexual satisfaction in women with breast cancer. 2017;14(1):S82. | |
|  |  | Abasher SM. The impact of psychological educational intervention program in improving psycho-sexual health of married women with breast cancer in khartoum state. Int J Gynecol Cancer. 2011;21(12 SUPPL. 3):S317. http://doi.org/10.1097/IGC.0b013e318235bd21 | |
|  |  | Abasher S. The impact of psychological intervention program in improving psycho-sexual health of married women with breast cancer in khartoum state. Support Care Cancer. 2016;24(1 Supplement 1):S104. http://doi.org/10.1007/s00520-016-3209-z | |
| Registration information or protocol | 26 | Reese JB, Zimmaro LA, Lepore SJ, Sorice KA, Handorf E, Daly MB, et al. Evaluating a couple-based intervention addressing sexual concerns for breast cancer survivors: study protocol for a randomized controlled trial. Trials. 2020;21(1):1-13. http://doi.org/10.1186/s13063-019-3975-2 | |
|  |  | NCT. BREast Cancer And Sexuality Treatment. 2020. | |
|  |  | NCT. RCT of an Internet-based CBT Program for Sexuality and Intimacy Problems in Women Treated for Breast Cancer. 2014. | |
|  |  | NCT. Addressing Sexual Concerns in Breast Cancer: patient Intervention Study. 2018. | |
|  |  | Narvaez A, Cortes-Funes F, Garcia A, Rubinos C, Gomez R. Evaluation of effectiveness of a group cognitive-behavioral therapy on body image, self-esteem, sexuality and distress in breast cancer patients. Psicooncologia. 2008;5(1):93-102. | |
|  |  | Hummel SB, van Lankveld JJDM, Oldenburg HSA, Hahn DEE, Broomans E, Aaronson NK. Internet-based cognitive behavioral therapy for sexual dysfunctions in women treated for breast cancer: design of a multicenter, randomized controlled trial. Bmc Cancer. 2015;15(1):1-12. http://doi.org/10.1186/s12885-015-1320-z | |
|  |  | Hummel SB, Van Lankveld J, Oldenburg H, Aaronson NK. Study protocol: a randomized study of an internet-based cognitive behavioural therapy program for sexuality and intimacy problems in women treated for breast cancer. 2013;22:257. http://doi.org/10.1111/j.1099-1611.2013.3394 | |
|  |  | RBR- P Qf. Pilates effect on sexuality, self-esteem and the quality of life of women who had Breast Cancer and survived. 2017. | |
|  |  | IRCT N. "Sexual Satisfaction in Breast Cancer". 2019. | |
|  |  | IRCT N. improving sexual function in breast cancer. 2019. | |
|  |  | IRCT N. Effect of sexual counseling on sexual satisfaction and function of women with breast cancer survival. 2020. | |
|  |  | IRCT N. Effect of illness perception on sexual satisfaction in breast cancer. 2018. | |
|  |  | IRCT N. Investigating the effect of sexual counseling by smartphone on sexual intimacy of women with breast cancer referring to Cancer Institute of Tehran Imam Khomeini Hospital2017-2018. 2018. | |
|  |  | IRCT N. Comparison of the effectiveness of the PLISSIT model with the Sexual health model on quality of life and sexual behavior among women with breast cancer. 2017. | |
|  |  | IRCT N. Effect of psychosexual counselling on the quality of sexual life of women with breast cancer. 2016. | |
|  |  | IRCT N. The impact of stress management consulting on sexual function and stress in women with breast cancer. 2016. | |
|  |  | IRCT N. The impact of telephone-based support on sexual function of women with breast cancer and their husband. 2018. | |
|  |  | IRCT N. The effect of supportive program on sexual self-concept of women with breast cancer. 2020. | |
|  |  | IRCT N. The effect of Cognitive Behavioral Therapy (CBT) on sexual function and satisfaction of women with breast cancer. 2019. | |
|  |  | IRCT N. The effect of counseling on body image and sexual satisfaction in women with breast cancer. 2017. | |
|  |  | IRCT N. Effect of counseling on body image and sexual satisfaction in women with breast cancer. 2016. | |
|  |  | IRCT N. The effect of virtual Solution Focused Counseling on Sexual quality of life of women Breast Cancer Survivors. 2021. | |
|  |  | IRCT N. The effect of counseling based of EXPLISSIT model on the sexual function of married women with lumpectomy for Breast cancer. 2018. | |
|  |  | IRCT N. Effect of consultation with the approach of motivational interview versus no consultation on sexual satisfaction and body image in women with breast cancer after mastectomy. 2016. | |
|  |  | IRCT N. The effect of group counseling based on problem solving on sexual quality of life in patients suffering from breast cancer in MRI center of Hamadan city in 2016. 2016. | |
|  |  | Han WH. Effect of extended nursing on quality of sexual life and quality of life in patients undergoing radical mastectomy. 2016. | |
| Unable to extract data | 12 | Xu B, Li SF, Liu YJ, Zhou JP, Wu DM. Effect of nursing intervention on quality of life of female breast cancer patients. Chin J Microecol. 2009;21:167-8. http://doi.org/10.1002/9780470611807.ch2 | |
|  |  | Wu YJ. Influence of nursing intervention on psychological and sexual life of patients with breast cancer after operation. Chin J Clin Ration Drug Use. 2017;10(3):145-6. http://doi.org/10.15887/j.cnki.13-1389/r.2017.03.071 | |
|  |  | Wang B, Wang YQ, Zhang SF, Zhang SY, Li BH. Effect of psychological intervention on negative emotions and sexual life quality of young and middle-aged women after radical mastectomy. Guide China Med. 2013;11(12):104-5. http://doi.org/10.3969/j.issn.1671-8194.2013.12.071 | |
|  |  | Sun TK. Effect of nursing intervention on psychology and sexual life of breast cancer patients after operation. Cardiovasc Dis J Integr Tradit Chin West Med. 2016;4(34):145. http://doi.org/CNKI:SUN:ZXJH.0.2016-34-116 | |
|  |  | Liu SQ. Application of wechat follow-up in sexual life guidance of breast cancer patients after surgery. Eat Well. 2017;4:132-3. http://doi.org/10.3969/j.issn.2095-8439.2017.05.156 | |
|  |  | Gao LX, Shang XQ, Wang ZL, Li CL, Zhang GL. Influence of psychological intervention on sexual function and marriage quality of breast cancer patients after accepting radical operation. Chin Nurs Res. 2011(4A):894-5. http://doi.org/10.3969/j.issn.1009-6493.2011.10.023 | |
|  |  | Gao BH. The value of cognitive intervention combined with family support in improving the sexual life quality of patients with breast cancer after childbearing age. J Community Med. 2018;16(10):69-71. http://doi.org/CNKI:SUN:SQYX.0.2018-10-029 | |
|  |  | Qiu L, Wang HL, He ZL. effect of psychological intervention based on personality traits on the operative psychological status and sexual life of breast cancer patients. Chin J Hum Sex. 2020;29(7):126-9. http://doi.org/10.3969/j.issn.1672-1993.2020.07.037 | |
|  |  | Oh B, Butow PN, Boyle F, Beale PJ, Costa D, Pavlakis N, et al. Effects of qigong on quality of life, fatigue, stress, neuropathy, and sexual function in women with metastatic breast cancer: a feasibility study. 2014;32(15 SUPPL. 1). | |
|  |  | Erol UF, Karayurt Ö. Effects of a Roy's Adaptation Model-Guided Support Group Intervention on Sexual Adjustment, Body Image, and Perceived Social Support in Women With Breast Cancer. Cancer Nurs. 2020. http://doi.org/10.1097/NCC.0000000000000854 | |
|  |  | Yuan QH. Effect of psychological intervention on psychological state and sexual life quality of 49 patients with breast cancer resection. J Guiyang Coll Tradit Chin Med. 2013;35(3):279-81. http://doi.org/10.3969/j.issn.1002-1108.2013.03.0140 | |
|  |  | Xu YE, Li J. Effect of cluster nursing on sexual life quality of patients after modified radical mastectomy for breast cancer. Int J Nurs. 2020;39(11):2077-80. http://doi.org/10.3760/cma.j.cn221370-20190125-00645 | |
| Undesirable language | 1 | Moon DH. Effect of Sexual Function Improvement Program for Breast Cancer Survivors on Sexual Distress, Sexual Satisfaction and Marital Intimacy. 2016;22(1):30-8. http://doi.org/10.4069/kjwhn.2016.22.1.30 | |
