# Supplementary figures and images for: Effectiveness of nursing interventions on the sexual quality of life of patients with breast cancer: A systematic review and meta-analysis
Source: PLoS One. 2022 Nov 3;17(11):e0277221. doi: 10.1371/journal.pone.0277221 (PMC9632802; doi:10.1371/journal.pone.0277221)

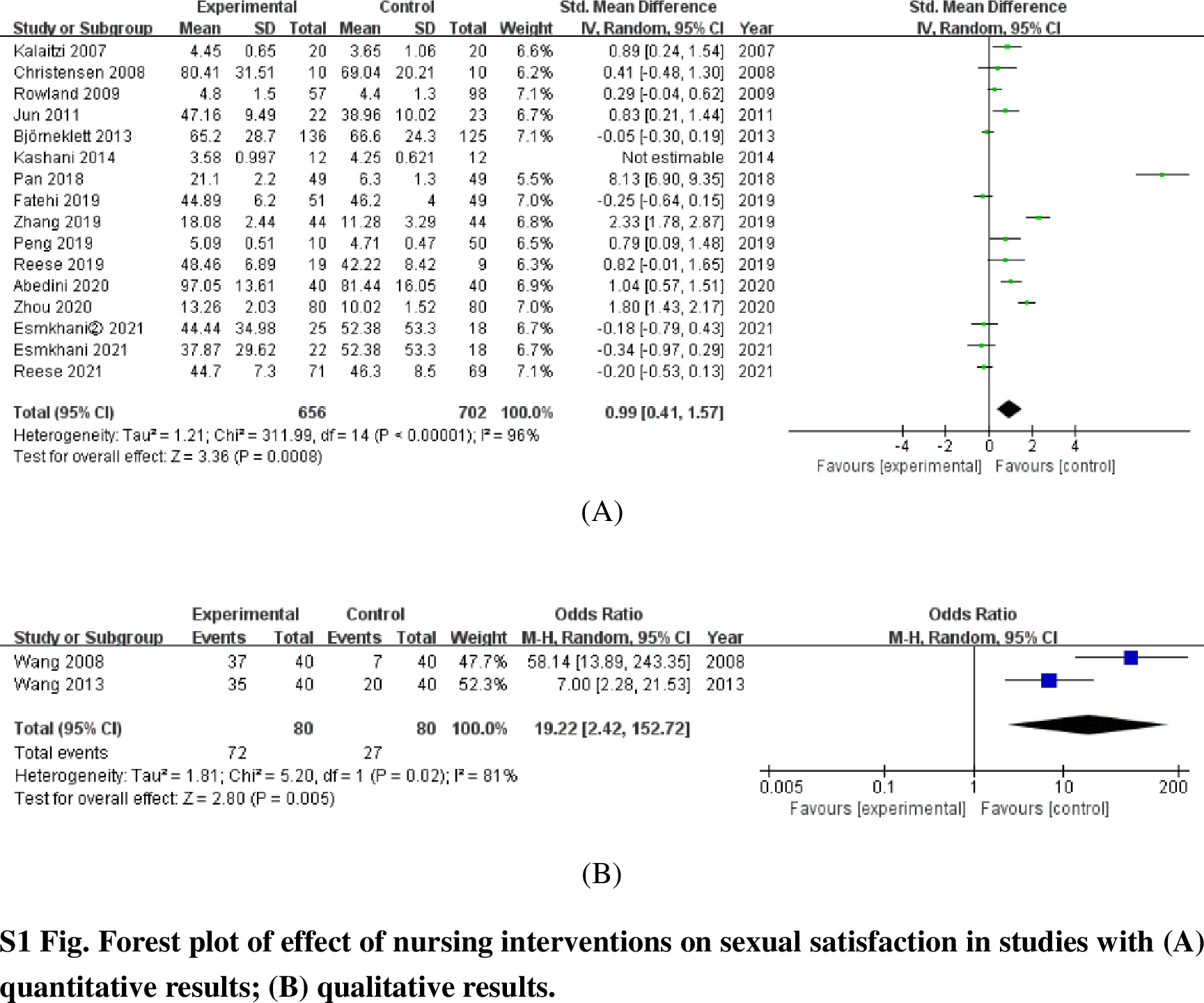

Supplement: S1 Fig — (TIF) [file pone.0277221.s005.tif]

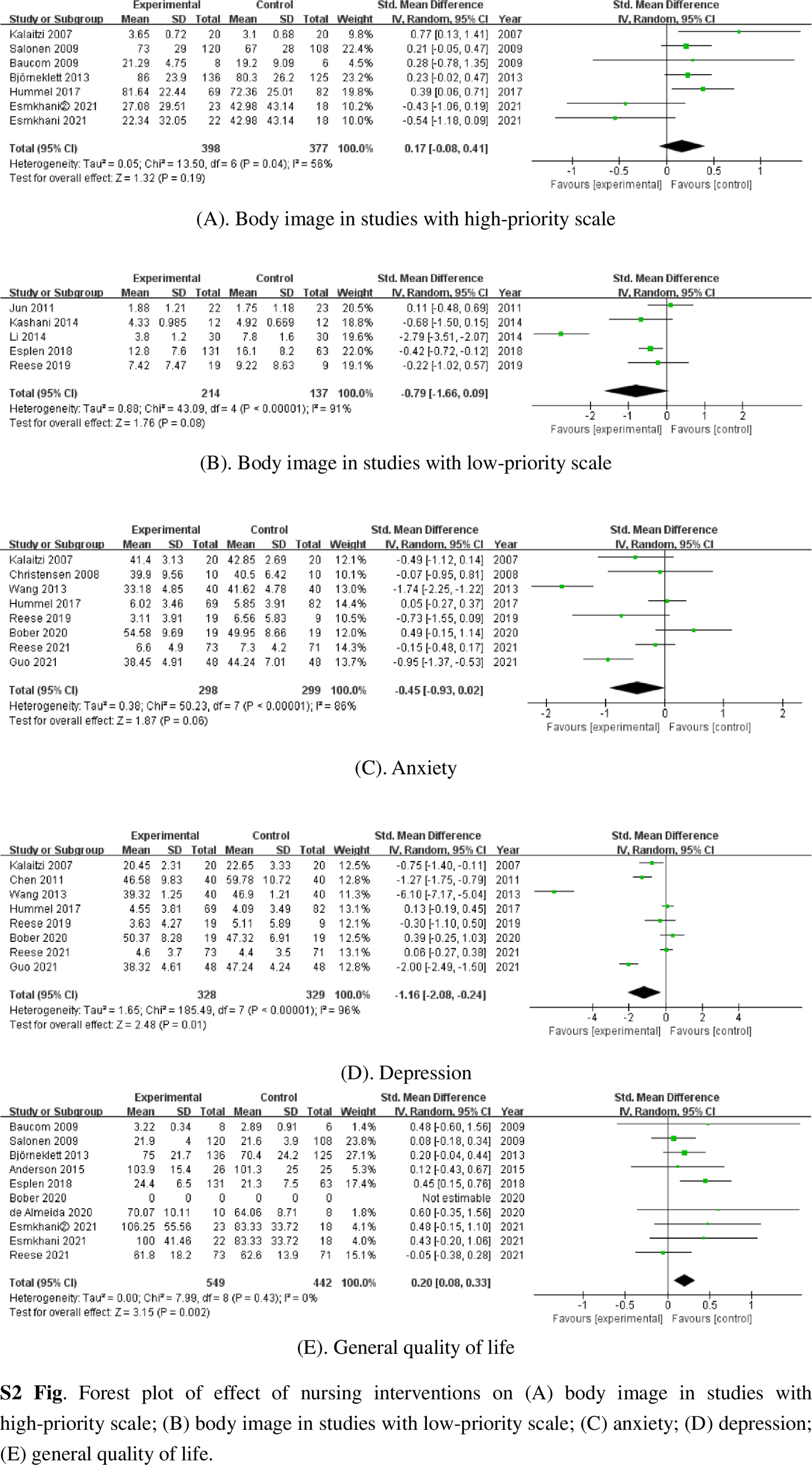

Supplement: S2 Fig — (TIF) [file pone.0277221.s006.tif]

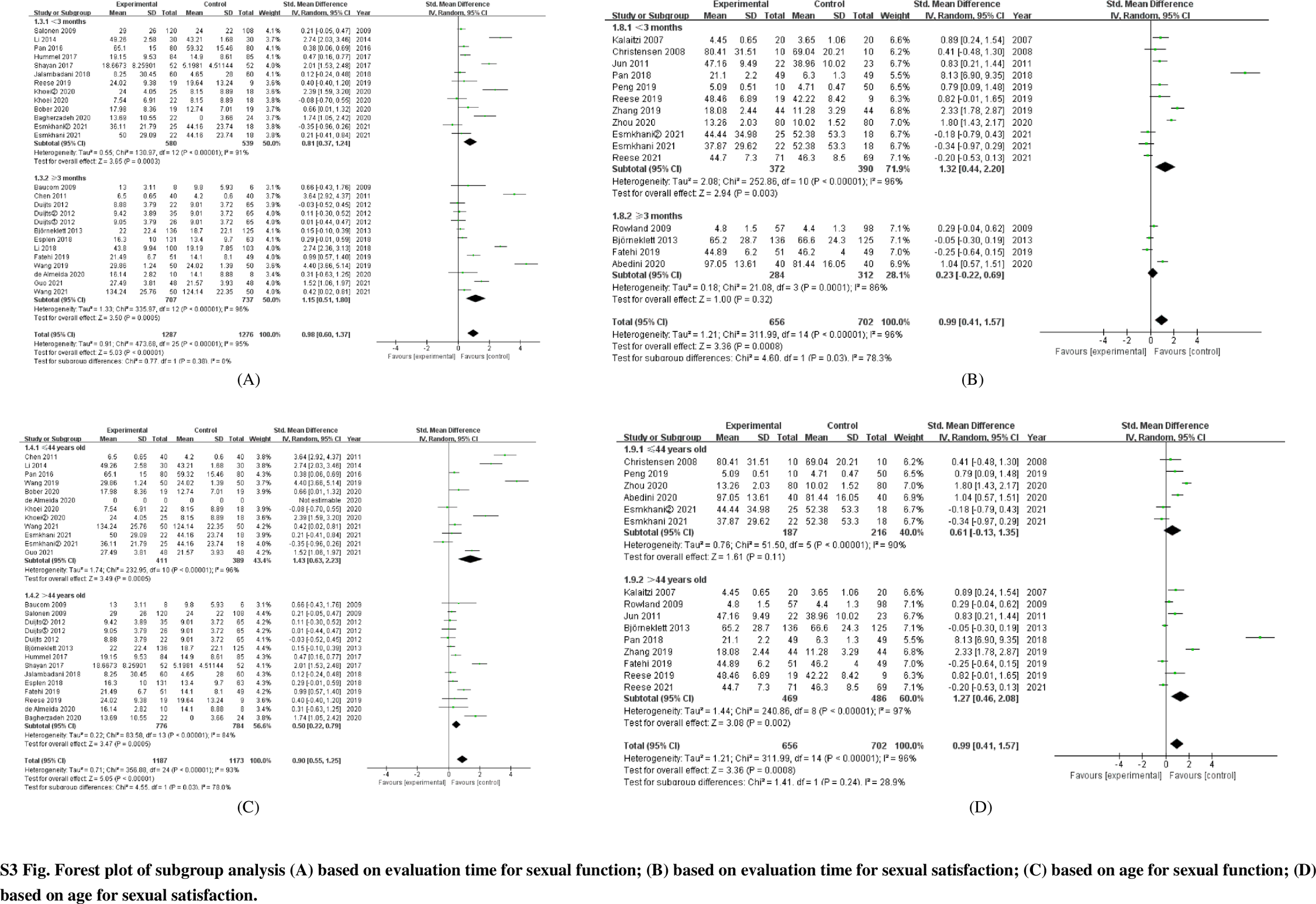

Supplement: S3 Fig — (TIF) [file pone.0277221.s007.tif]
